# PKU dietary handbook to accompany PKU guidelines

**DOI:** 10.1186/s13023-020-01391-y

**Published:** 2020-06-30

**Authors:** A. MacDonald, A. M. J. van Wegberg, K. Ahring, S. Beblo, A. Bélanger-Quintana, A. Burlina, J. Campistol, T. Coşkun, F. Feillet, M. Giżewska, S. C. Huijbregts, V. Leuzzi, F. Maillot, A. C. Muntau, J. C. Rocha, C. Romani, F. Trefz, F. J. van Spronsen

**Affiliations:** 1 Dietetic Department, Birmingham Children’s Hospital, Birmingham, UK; 2 Division of Metabolic Diseases, Beatrix Children’s Hospital, University Medical Centre Groningen, University of Groningen, Hanzeplein 1, 9700 RB Groningen, The Netherlands; 3 Department of PKU, Kennedy Centre, Glostrup, Denmark; 4 Department of Women and Child Health, Center for Pediatric Research Leipzig, Hospital for Children and Adolescents, University Hospitals, Leipzig, Germany; 5 Metabolic Diseases Unit, Department of Paediatrics, Hospital Ramon y Cajal Madrid, Madrid, Spain; 6 Division of Inherited Metabolic Diseases, Department of Paediatrics, University Hospital of Padova, Padova, Italy; 7 Neuropaediatrics Department, Hospital Sant Joan de Déu, Universitat de Barcelona, Barcelona, Spain; 8 Hacettepe University Faculty of Medicine, Ankara, Turkey; 9 Department of Paediatrics, Hôpital d’Enfants Brabois, CHU Nancy, Vandoeuvre les Nancy, France; 10 Department of Paediatrics, Endocrinology, Diabetology, Metabolic Diseases and Cardiology of the Developmental Age, Pomeranian Medical University, Szczecin, Poland; 11 Department of Clinical Child and Adolescent Studies-Neurodevelopmental Disorders, Faculty of Social Sciences, Leiden University, Leiden, The Netherlands; 12 Department of Paediatrics, Child Neurology and Psychiatry, Sapienza University of Rome, Via dei Sabelli 108, 00185 Rome, Italy; 13 CHRU de Tours, Université François Rabelais, INSERM U1069, Tours, France; 14 University Children’s Hospital, University Medical Centre Hamburg-Eppendorf, 20246 Hamburg, Germany; 15 Nutrition & Metabolism, NOVA Medical School, Faculdade de Ciências Médicas, Universidade Nova de Lisboa, Lisbon, Portugal. Centre for Health Technology and Services Research (CINTESIS), Porto, Portugal; 16 School of Life and Health Sciences, Aston University, Birmingham, UK; 17 Department of Paediatrics, University of Heidelberg, Heidelberg, Germany

**Keywords:** Phenylketonuria, PKU, Diet, Treatment, Recommendations, Guidelines

## Abstract

**Background:**

Phenylketonuria (PKU) is an autosomal recessive inborn error of phenylalanine metabolism caused by deficiency in the enzyme phenylalanine hydroxylase that converts phenylalanine into tyrosine.

**Main body:**

In 2017 the first European PKU Guidelines were published. These guidelines contained evidence based and/or expert opinion recommendations regarding diagnosis, treatment and care for patients with PKU of all ages. This manuscript is a supplement containing the practical application of the dietary treatment.

**Conclusion:**

This handbook can support dietitians, nutritionists and physicians in starting, adjusting and maintaining dietary treatment.

## Background

Phenylketonuria (PKU) is an autosomal, recessive, genetic disorder. It is caused by a deficiency of the enzyme phenylalanine hydroxylase which normally converts phenylalanine to tyrosine. Deficiency of this enzyme leads to an increased production of phenylketone bodies (hence phenylketonuria) and accumulation of phenylalanine resulting in high phenylalanine levels in the blood and brain.

A strict, lifelong low phenylalanine diet is the principle treatment in PKU. It may be the only treatment or used in combination with drug treatments. This diet manual describes the practical application of the European PKU guidelines [1]. It provides additional references supporting the practical recommendations as listed in the European PKU Guidelines [1].

## Principles of dietary management

The goals of dietary management are threefold:

- Prevent accumulation of excessive phenylalanine in the blood (and therefore the brain) by strict control of natural protein/phenylalanine intake.
- Replacement of natural protein that has been removed from the diet with safe or phenylalanine-free protein, called synthetic protein, amino acid mixture/supplement or protein substitute. All protein substitutes are phenylalanine-free or very low in phenylalanine.
- Attainment of normal growth and nutritional status. This is achieved by ensuring that the diet contains a balanced intake of all nutrients and energy. Vitamins and minerals supplements are either added to the protein substitute or given as a separate supplement.

## Foods avoided in a low phenylalanine diet

In most people with PKU, the natural protein or dietary phenylalanine is restricted to 25% or less of a regular intake to maintain blood phenylalanine concentrations within European PKU Guidelines target ranges [1].

This usually requires avoidance/restriction of all high protein foods such as:

- meat, chicken, fish, eggs, cheese derived from animal milk (cow, goat, sheep).
- nuts, seeds, quinoa, wheat, oats, rye, barley.
- foods made from Quorn™ (a meat substitute made from protein of fungal origin).
- soya, tempeh, pulses/lentils.
- gelatin and plant algae such as spirulina.
- aspartame (sweetener).

## The difference between phenylalanine and natural protein

Phenylalanine is an amino acid found in natural protein. Different foods contain different amounts of phenylalanine. In animal (e.g. meat, fish, milk and eggs) and cereal protein sources (e.g. wheat flour and breakfast cereal), usually the amount of food that is calculated to provide 1 g protein will supply approximately 50 mg phenylalanine. Animal and cereal proteins contain around 5% phenylalanine. *This means that the phenylalanine content of these foods can be estimated from the food protein labelling, without knowing the phenylalanine content.*

However, fruits and vegetables usually contain a lower and more variable phenylalanine content, between 20 to 40 mg/per 1 g protein. Consequently the phenylalanine content of fruit and vegetables cannot be calculated accurately from the food nutritional analysis label on a packet/container that only declares the protein content. The protein content may give the impression these foods are higher in phenylalanine than they are (exceptions include spinach, peas, seaweed, kale and sweetcorn which have a higher phenylalanine/protein ratio).

Many fruits and vegetables have been analysed specifically for their phenylalanine content [2–4].

## Phenylalanine tolerance

Phenylalanine tolerance is the amount of phenylalanine that can be eaten by an individual with PKU whilst maintaining blood phenylalanine concentrations within the target treatment range. Target blood phenylalanine ranges are: 120 to 360 μmol/L for children up to the age of 12 years of age and women on a pre-conception diet or pregnancy; and 120 to 600 μmol/L for patients ≥12 years of age.

The amount of dietary phenylalanine tolerated will vary between individuals depending on the severity of each person’s PKU (people with mild or moderate PKU will tolerate more protein), the dosage, adherence and daily distribution of protein substitute, or if the drug therapy, sapropterin or Pegvaliase for (patients ≥16 of age) is part of the treatment regimen. It is also influenced by growth, pregnancy, and catabolic state during illness. Most patients on diet treatment tolerate less than 500 mg/day phenylalanine [5]. It is expected that patients who are responsive to sapropterin should at least double their phenylalanine tolerance or tolerate a safe level of protein intake as defined by the WHO/FAO/UNU 2007 [6, 7].

## Establishing maximum phenylalanine tolerance

Initial phenylalanine tolerance is established in early infancy, with the amount of breast milk or standard infant formula being titrated with the blood phenylalanine levels. After this time, phenylalanine intake is commonly only adjusted (increased/decreased) if blood phenylalanine levels are consistently outside the target range.

In practice, it is better for individuals if their phenylalanine is maximized according to individual tolerance. The more phenylalanine that is tolerated, the more acceptable the diet will be, and it will ease the practical and social burden that the diet demands. Additionally, more natural protein will bring nutritional benefits. Physiologically, natural nutrients are more likely to be more efficiently utilized.

There is evidence to suggest that some individuals with PKU may tolerate more phenylalanine than they have been prescribed by their health professionals [8–10]. The only way that this can be tested is to systematically challenge individuals with additional phenylalanine. It is particularly important that patients fully adhere with their prescribed dose of protein substitute as this will help stimulate protein synthesis.

If blood phenylalanine levels are consistently maintained within the *lower* half of target blood phenylalanine levels for at least 3 months (i.e. 120 to 240 μmol/L in children up to 12 years of age and 120 to 360 μmol/L if aged ≥12), an increase of phenylalanine intake by an additional 50 mg/day (approx. 1 g natural protein) should be considered. If blood phenylalanine levels remain within the lower half of the target blood phenylalanine range for a further 3 consecutive blood phenylalanine levels, consideration should be given to repeating this process.

If blood phenylalanine levels increase above target range, then additional phenylalanine should be removed, and the patients should return to the original prescribed amount of phenylalanine.

These dietary changes should always be done under the supervision of a metabolic dietitian or physician.

## Phenylalanine allocation in the diet

All individuals with PKU should be allocated a daily allowance of phenylalanine according to their individual tolerance. This enables individuals to maintain a consistent phenylalanine/protein intake. There are different ways of calculating phenylalanine/protein intake and there is little evidence to show that any method is better than another. Phenylalanine/protein intake may be calculated by daily grams of protein or milligrams of phenylalanine. Throughout Europe, health professionals may use one of five systems to calculate the amount of phenylalanine eaten each day and all systems achieve acceptable blood phenylalanine control. In all systems, fruits and vegetables **except** those containing phenylalanine ≥75 mg/100 g can be given without estimating their individual phenylalanine content. This means they are not calculated as part of the daily phenylalanine analysis. One exception is potatoes, although potato varieties mainly contain ≤75 mg/100 g, the potential amount that may be eaten each day is high, so they are calculated/measured within the daily allowance of phenylalanine.

The 5 systems for calculating phenylalanine intake are:

- Phenylalanine exchange system (e.g. 50, 25, 20 mg of phenylalanine).
- 1 g protein exchanges.
- 1 g protein exchanges (but using the phenylalanine analyses of fruit and vegetables and calculating the weight that provides 50 mg phenylalanine).
- Paper lists/apps listing the phenylalanine content of foods.
- Paper lists/apps listing the protein content of foods.

Examples of how to calculate the phenylalanine in each system are given in Appendix 1.

For regular manufactured foods e.g. sauces, breakfast cereals, their protein food labelling is used to determine if they are suitable for a low phenylalanine diet. This can be challenging as sometimes protein labelling is confusing. Specific considerations when using protein labelling are given in Table 1.

**Table 1:** Considerations when interpreting protein analysis from food labels

• It is protein rather than phenylalanine content stated on food nutrition labels of regular foods. • For foods produced in the EU, if the protein content is ≤0.5 g/100 g, the food product can state it contains 0 g protein. • The USA consider a protein content of < 1 g is insignificant and it will not state the specific amount of protein per serving size if the protein content is < 1 g/portion size. • The protein content on a food label may not differentiate between cooked or uncooked weight. • In dry foods, protein content may be listed only after a food has been ‘theoretically’ reconstituted. Manufacturers may assume that some food items are reconstituted with cow’s milk e.g. desserts or custards and this is the protein value that may be stated on the label. This will overestimate the protein content if dry foods are reconstituted with low protein milk rather than cow’s milk.	

## Adapting phenylalanine intake if blood phenylalanine levels are higher than recommended

No studies have been conducted to give an indication of how much dietary change is needed when blood phenylalanine levels have been consistently above target range for many years. The degree of change necessary may vary from individual to individual and will also be influenced by the presence of catabolism, growth and pregnancy. It may be that for some patients, strict adherence with the existing dietary prescription is enough to achieve the European PKU Guidelines target ranges. Other patients may need a significant reduction in their phenylalanine intake.

Several steps may be necessary to achieve blood phenylalanine concentrations within target recommendations:

- Carefully document all intake of food, drink and protein substitute. Any dietary adjustment should be supervised by the health care team.
- The following course of action is recommended depending on dietary history findings:
  i. If a patient does not fully adhere to their usual dietary regimen, try a 4-week trial of the ‘usual’ prescribed ‘diet’ and this should give an indicator of what level of blood phenylalanine control can be achieved.
  ii. If a patient is eating an unrestricted diet (i.e. meat, fish and eggs), it may be enough to remove some or all high protein foods from the diet and supplement with a suitable protein substitute to bring blood phenylalanine to within target range. Overall, the protein intake should supply 40% more than the FAO/WHO/UNU [7] safe levels of protein intake accounting for both natural protein intake and protein equivalent from protein substitute [1].
  iii. If blood phenylalanine levels remain higher than the target ranges, the next stage is to gradually decrease the dietary phenylalanine by 50 mg/day (equivalent to 1 g protein) at each diet adjustment. If after 3 consecutive blood spots, phenylalanine levels are not within target range, repeat this step and reduce dietary intake by a further 50 mg/day (protein, 1 g/day), until blood phenylalanine levels are within target range. Dietary phenylalanine intake should not be reduced to less than a minimum of 150 mg/day in children under regular conditions [5]. The minimum phenylalanine intake for adults with PKU remains undefined.
- During any dietary change it is important to monitor blood phenylalanine levels regularly. Weekly blood spots are recommended.

## Low protein foods

Giving adequate energy intake from very low protein sources is essential to meet energy requirements and to minimise h catabolism that can lead to poor blood phenylalanine control. Maintenance of a normal energy intake is achieved by encouraging: 1) use of ‘regular’ foods which are very low in protein and 2) special manufactured low protein foods such as bread and pasta (Table 2).

**Table 2:** Low protein foods that can be eaten without restriction in a low phenylalanine diet

Food groups	Examples of low protein foods that can be eaten without calculation or restriction in a low phenylalanine diet
Fruits and vegetables	Fruits and vegetables containing phenylalanine ≤ 75 mg/100 g. The exception to this rule is potatoes, which are calculated and measured within the phenylalanigne exchange system.
Fats	Butter, margarine, ghee, and vegetable oils. Generally, any fat spread containing protein ≤ 1 g/100 g can be given in the diet without calculating its phenylalanine/protein content [11].
Starches	Cassava flour, arrowroot, sago, tapioca, and corn starch that contains protein ≤0.5 g/100 g (phenylalanine content ≤ 25 mg/100 g).
Vegan cheese	Any vegan cheese that contains protein ≤ 0.5 g/100 g (or ≤ 25 mg phenylalanine/100 g) can be given in the diet without calculating the phenylalanine/protein content. If it contains > 0.5 g/100 g (or > 25 mg phenylalanine /100 g), it should be measured/calculated in the phenylalanine/natural protein allowance.
Sugars	Sugar, glucose, jam, honey, marmalade, golden syrup, maple syrup, fruit sorbets, ice lollies, and sweets that contains protein ≤ 0.5 g/100 g (phenylalanine content ≤ 25 mg/100 g) [11].
Vegetarian jelly /agar agar (gelatin free)	If jelly/agar contains protein ≤ 0.5 g/100 g (or phenylalanine ≤ 25 mg/100 g) it can be given in the diet without restriction. If it contains protein > 0.5 g/100 g (> 25 mg phenylalanine /100 g, it should be measured/calculated in the phenylalanine/natural protein allowance.
Low protein special foods	A selection of low protein breads, flour mixes, pizza bases, pasta, biscuits, and egg replacers, are available. Low protein special products are allocated in the diet without restriction if all ingredients are exchange-free e.g. food starch and oil. If they contain protein containing ingredients and contain > 25 mg phenylalanine/100 g, their phenylalanine should be calculated in the diet.
Herbs/spices	All herbs, spices and seasonings can be incorporated into the diet without phenylalanine calculation as the quantity used in cooking is very small.
Drinks	Water, squash, lemonade, cola drinks, fruit juice, black tea, fruit tea, green tea, coffee, tonic water, soda water and mineral water are all permitted providing they are aspartame free.
Low protein/low Phe special milk	Any low protein special milk replacements that provide a total phenylalanine intake of > 25 mg over 24 h in the volume consumed should be calculated/measured in the phenylalanine allowance. If the total phenylalanine intake from low protein milk replacements provides phenylalanine ≤25 mg over 24 h, it can be given without restriction [11].
Plant milk	Any plant milk (e.g. coconut, rice, almond) that contains protein > 0.1 g/100 ml should be calculated/measured in the diet.
Miscellaneous	Food essences and food colouring are used in small quantities and can be given without restriction.

There are many regular foods which are naturally very low in protein that can be eaten without measurement. These include vegan cheese made from oils and starch (containing protein ≤0.5 g/100 g or phenylalanine ≤25 mg/100 g), butter, margarine, vegetables oils, low protein starches (tapioca, arrowroot, corn starch, cassava flour), sugar, jams, and honey.

Although all fruits and vegetables containing phenylalanine ≤75 mg/100 g contribute a small amount of daily phenylalanine, this is generally not enough to affect blood phenylalanine control. These fruit and vegetable sources are not calculated in the daily phenylalanine allowance (list of suitable fruits and vegetable in Table 3), and are given without restriction.

**Table 3:** Fruits and vegetables allowed without measurement in PKU

**Fruit**		
Fresh, frozen, or tinned in syrup (≤75 mg phenylalanine /100 g)		
Apple	Kiwi fruit	Pomegranate
Apricots	Kum quats	Prickly pear
Bananas	Lemon	Prunes
Bilberries	Limes	Quince
Blackberries	Loganberries	Raisins
Blackcurrants	Lychees	Raspberries
Blueberries	Mandarins	Redcurrants
Clementines	Mango	Rhubarb
Cherries	Melon	Satsumas
Cranberries	Medlars	Sharon fruit
Currants, dried	Mulberries	Star fruit
Damsons	Nectarines	Strawberries
Dragon fruit	Olives	Sultanas
	Oranges	Tamarillo
	Paw paw	Tangerine
Gooseberries	Peaches	Watermelon
Grapefruit	Pears	Mixed peel
Grapes	Physalis	Angelica
Greengages	Pineapple	Glace cherries
Guava	Plantain	Ginger
Jack fruit	Plums	
**Vegetables**		
Fresh, frozen or tinned vegetables (≤75 mg phenylalanine /100 g)		
Avocado	Eddoes	Pak choi
Artichoke	Endive	Parsnip
Aubergine	Fennel	Peppers
Baby corn	Garlic	Pumpkin
Beans – French, green	Gherkins	Radish
Beetroot	Gourd	Samphire
Cabbage	Karela	Spring onion
Carrots	Kohl rabi	Squash: acorn, butternut
Capers	Ladys finger (Okra)	Swede
Cassava	Leeks	Sweet potato
Celeriac	Lettuce	Tomatoes
Celery	Marrow	Tomato puree
Chayote	Mooli	Turnip
Chicory	Mustard and cress	Watercress
Courgette	Onions	Water chestnuts
Cucumber	Parsley and all herbs	
Dudhi	Parsnips	

Table adapted from MacDonald A, White F. Amino acid disorders. In Shaw V, editor. Clinical Paediatric Dietetics: Chichester: Wiley Blackwell; 2015. p. 430

Potatoes are an exception (see Phenylalanine allocation in the diet section). Also, cooked crisps (snacks) made from vegetables such as beetroot or parsnips are concentrated in phenylalanine and should be calculated/measured in the daily phenylalanine allowance.

Although there is a wide range of ‘starch based’ specially manufactured low protein foods such as bread, flour, pasta and biscuits available, patients have variable access to these across Europe. They are generally expensive if not government/insurance funded or supplied by the hospitals. They may provide up to 50% of energy intake. Patients using sapropterin have less need for special low protein foods.

## Protein substitutes

The provision of an adequate dose of protein substitute, usually based on phenylalanine-free amino acids supplements, is essential to promote normal growth, prevent protein deficiency, provide a source of tyrosine, and help optimise blood phenylalanine control [1]. For people with classical PKU, the protein substitute is likely to supply at least 75% of daily nitrogen requirements.

The European PKU Guidelines recommend that the total protein intake should supply 40% more than the FAO/WHO/UNU safe levels of protein intake [1]. However, this amount is arbitrary and unconfirmed by research. As most of the available protein substitutes are derived completely from phenylalanine-free amino acid sources, it is recommended that a dose higher than the FAO/WHO/UNU is given. This extra amount compensates for the ineffective absorption of natural/intact protein (which is mainly plant based), poor utilisation of L-amino acids and sub-optimal energy intake [12, 13]. Body weight, age, growth and the prescribed amount of phenylalanine/natural protein are considered when determining the dose of protein substitute. If an individual with PKU is obese, the protein substitute requirement should be based on ideal body weight (although this is an arbitrary measure, it is probably the most practical).

### Timing and distribution of protein substitute intake

It is advised to give the protein substitute in small frequent doses, 3–4 times evenly throughout the day, rather than once or twice-daily [14]. This should be taken together with natural protein and a carbohydrate source [15].

### Types of protein substitute

Protein substitutes are in the form of amino acid powders, capsules, tablets, bars and liquids and may contain added carbohydrate, fat, vitamins and minerals. The general types and features of protein substitutes are outlined in Table 4. Protein substitute adherence is an issue and it is important that a choice of age and nutritionally appropriate types and presentations are offered.

**Table 4:** Types of protein substitutes available based on phenylalanine-free amino acids

Infant protein substitutes (powder)	Powdered infant formula without phenylalanine has a similar nutritional composition to regular formula for infants without PKU.
Infant protein substitutes (liquid)	Liquid infant formula without phenylalanine has a similar nutritional composition to regular formula for infants without PKU.
Powdered weaning protein substitutes	Powdered weaning (thickened) amino acids without phenylalanine. The semi-solid consistency can be adjusted to suit the developmental age of the older baby/toddler.
Semi-solid weaning protein substitutes	Ready to use semi-solid protein substitutes (amino acids mixed with fruit puree) for older babies/toddlers.
Powdered protein substitutes	Powdered amino acid supplements (with or without the addition of vitamins, minerals, long chain fatty acids, carbohydrate and fat), reconstituted with water and made into semi-solid (spoonable) consistency or drink. They are suitable for variable age groups and are available in different flavours.
Liquid protein substitutes	Ready to use liquid protein substitutes (usually with the addition of vitamins, minerals, long chain fatty acids, carbohydrate and fat). They are suitable for variable age groups and are available in different flavours.
Protein substitute tablets and capsules	Tablets or capsules containing amino acids without phenylalanine ±vitamins and minerals. Usually high numbers of capsules or tablets are required to meet full protein substitute requirement, but they can be used in combination with other protein substitutes to make up requirements and aid variety.
Protein substitute bars	Snack bars containing amino acids without phenylalanine. They are usually given in combination with other protein substitutes to make up requirements and aid variety. Some may not contain added vitamins and minerals.

### Administration of protein substitute

- Protein substitutes based on amino acids have a high osmolality. Osmolality is the concentration of a solution expressed as the total number of solute particles per kilogram. Products with a high osmolality may lead to delayed gastric emptying and cause diarrhoea. Therefore, it is recommended that extra water is given with each dose of protein substitute, particularly if they are given more concentrated than recommended by the manufacturer.
- When water is added to a powdered protein substitute, the tyrosine is hydrophobic and forms an insoluble layer at the top of the solution making the product less acceptable. Using a shaker beaker should produce a homogenous mixture.
- Ideally, all protein substitutes should be prepared immediately prior to use. Some preparations contain starch that thickens with time.
- It is essential that protein substitute supplies are carefully rotated within the home and stored according to manufacturer’s instructions. Protein substitute quality may be less acceptable if it is close to its shelf life date or stored in warm conditions.

### Casein glycomacropeptide (CGMP)

This is a low phenylalanine protein that is used as a protein substitute in PKU. It is a by-product of cheese whey, and although theoretically phenylalanine-free, due to the extraction process, some residual phenylalanine remains in the manufactured product. CGMP protein substitutes (± vitamins and minerals) are available in powdered, liquid and bar presentations.

Research has reported potential advantages of using CGMP in PKU compared with Phe-free amino acid supplements. In humans, there is evidence that CGMP has a better taste, improves satiety and improves nitrogen retention. In animal (PKU mice) studies, there is suggestion that CGMP reduces blood phenylalanine in the brain, improves bone health and acts as prebiotic [16].

One supplier provides most of the CGMP protein; this provides 1.8 mg phenylalanine for each 1 g protein equivalent [17]. Taking 60 g/day of *low Phe* protein from a CGMP protein substitute (the average adolescent /adult dose) will give an extra 108 mg/day phenylalanine. The European PKU Guidelines requires further evidence before it can give practical recommendations on the use of CGMP in PKU [1]. However, it is now established that CGMP, when given for the entire dose of protein substitute, increases blood phenylalanine levels in well treated children with PKU, so care should be taken when replacing amino acids with CGMP [18]. Nevertheless, children with milder forms of PKU or on sapropterin therapy with a higher natural protein tolerance should tolerate the extra phenylalanine provided by CGMP. No significant increase in blood phenylalanine has been observed in adult patients using CGMP [16], although some patients may have had sub-optimal blood phenylalanine control prior to CGMP commencement or they may have received only a partial amount of their protein substitute intake from this source.

## Aspartame content of foods/drinks

Aspartame (E951) is an artificial sweetener and 56% is converted to free phenylalanine and so should be excluded in a low phenylalanine diet. It is added to soft drinks, chewing gums, sweets, desserts, jelly and tabletop sweeteners. Although aspartame should always be included on food labels, the amount of phenylalanine it provides remains undisclosed. The European Commission 1129/2011 issue maximum safe levels of aspartame that can be added to individual categories of foods and drinks. The types of foods that may contain phenylalanine from aspartame with the maximum amount per litre or kg of food is given in Appendix 2.

Aspartame is added to some medications. The European Medicines Agency summary of product characteristics may identify the amount of phenylalanine from aspartame added to a specific medication.

Neotame contains aspartame but the production of phenylalanine is limited due to the inability to break down the peptide bond between aspartic acid and phenylalanine, reducing the production of phenylalanine. This sweetener is safe in PKU, but is more expensive, and therefore is used less by the industry.

Safe sweeteners for PKU are listed in Table 5.

**Table 5:** Sweeteners and other sugar alternatives suitable in PKU

•Acesulfame K – E950 •Saccharin – E954 •Steviol glycosides •Sucralose- E955 •Fructose •Sucrose •Maltodextrin •Mannitol •Sorbitol •Xylitol	

## Key points for a balanced diet/healthy living with PKU (Fig. 1)

A balanced diet for a person with PKU should consist of the following:

- Taking the prescribed dose of protein substitute divided into at least 3 equal doses spread throughout the day. The protein substitute is usually supplemented with all vitamins, minerals and ± long chain fatty acids to meet nutritional requirements. If vitamins, minerals and long chain fatty acids (e.g. docosahexaenoic acid) are not supplied by the protein supplement, then additional supplementation should be provided. If there is poor adherence with the protein substitute, then additional vitamins and minerals may be required and biochemical nutritional status should be carefully monitored, particularly vitamin B 12.
- Phenylalanine intake should be divided throughout the day.
- Encourage the use of fruit and vegetables containing phenylalanine ≤75 mg/100 g. Try and give 5 portions/day with at least one portion at each meal. A portion of fruits and vegetables is defined as a handful (using the patient’s hand size as a guide).
- Encourage special low protein foods such as bread and pasta at most meals to provide calories, aid satiety, and variety.
- Encourage additional water with protein substitute.
- Monitor blood phenylalanine levels regularly (see European PKU guidelines for frequency), at the same time of day but preferably fasting.
- Visit dental health practitioners annually and maintain good oral dental hygiene practices.

**Fig. 1:**
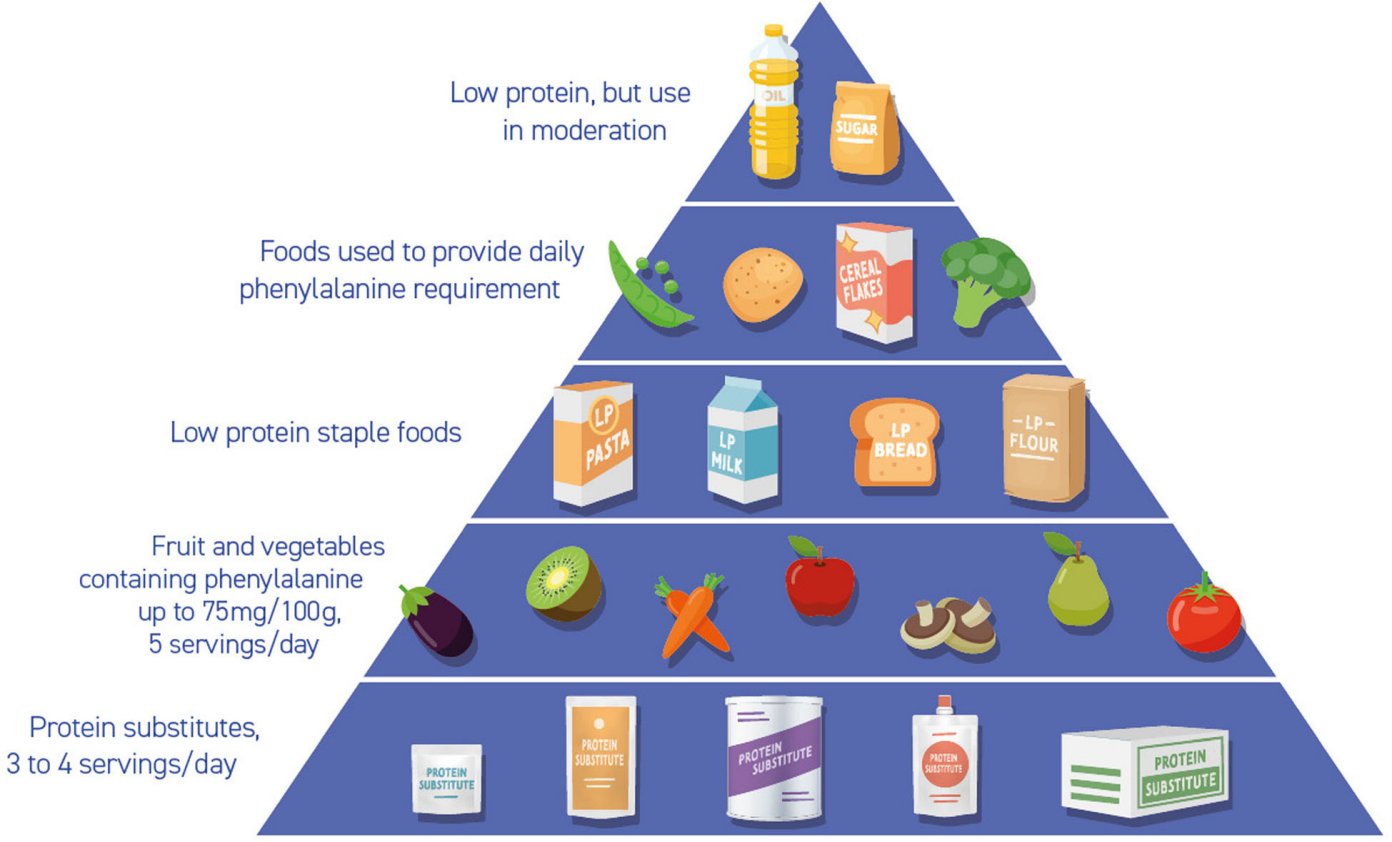
Food pyramid for PKU

## Dental care

There are few studies that report the prevalence of dental caries in PKU with results of studies contradictory. There are certainly conflicting objectives in PKU care, i.e. the need to maintain optimal blood phenylalanine control and a low phenylalanine diet involves eating a relatively high carbohydrate intake with a phenylalanine-free protein substitute that is acidic and sweetened and taken three to four times daily.

To help prevent dental problems in PKU, the following should be encouraged:

- Rinse the mouth with water after taking protein substitute.
- Drink water instead of juice throughout the day.
- Use a toothpaste containing fluoride.
- Encourage a good dental health routine.
- Schedule regular check-ups with a dentist from an early age.
- After the age of 6 months, infant drinks should be offered in a non-valve free flowing cup.
- Juices or sugary drinks should not be added to infant bottles; protein substitutes should not be added to infant bottles > 1 year of age. Discourage infants from sleeping with a bottle or beaker in their mouth.
- Sweet foods and juices are better confined to mealtimes only. Avoid using dummies/pacifiers from 12 months of age. Using dummies after this age encourages an open bite (which is when teeth move to make space for the dummy).

## Interpreting blood phenylalanine levels and potential dietary adjustment

Tables 6 and 7 provide an overview of causes of high and low blood phenylalanine levels, including appropriate actions to take.

It is better to monitor large space trends in blood phenylalanine levels before increasing/ decreasing natural protein/phenylalanine intake.

**Table 6:** High blood phenylalanine levels and suggested actions

Cause of high blood phenylalanine levels	Action
Fever/infection/trauma	See section on illness management
Excess natural protein intake	• Check understanding/calculation of phenylalanine allowance/ exchanges, misinterpretation/misunderstandings of protein amounts in foods. Review portion sizes. • Check any intentional dietary non-adherence (e.g. patient chose to eat extra protein). • Check any special low protein products are low protein and not gluten-free by accident. • Re-educate patient or family if necessary.
Inadequate intake of protein substitute	• Check adherence (at home, nursery, school). Explore any reasons for poor adherence and either re-educate or change type or flavour of protein substitute if appropriate. • Check timing of protein substitute (should be spread throughout the day). • Check patient has adequate supply of protein substitute. • Re-calculate dose of protein substitute and increase the dosage if necessary.
Incorrect prescription of protein substitute	• Occasionally the wrong protein substitute may accidentally be prescribed or given by the pharmacist or a home delivery company.
Low energy intake/weight loss/catabolism	• Increase energy intake/give extra carbohydrate. Encourage extra low protein foods or high calorie drinks.
No obvious reason	• If blood phenylalanine levels are consistently high, consider a reduction in natural protein/phenylalanine by approx. 0.5 to 1 g/day protein or 25 to 50 mg phenylalanine/day.

Table adapted from MacDonald A, White F. Amino acid disorders. In Shaw V, editor. Clinical Paediatric Dietetics: Chichester: Wiley Blackwell; 2015. p. 433

**Table 7:** Low blood phenylalanine levels and action that should be taken

Cause of low blood phenylalanine levels	Action
Inadequate intake of natural protein	• Ensure all prescribed intake of natural protein/phenylalanine is eaten. • Check understanding of exchange system/phenylalanine content of foods. • Re-educate as necessary.
Anabolic phase, following an intercurrent infection	• Ensure all prescribed intake of natural protein is eaten. • Repeat blood phenylalanine level, and if is still low, consider an increase of natural protein by approx. 0.5-1 g protein or 25 to 50 mg/day phenylalanine but monitor blood phenylalanine levels carefully.
Rapid growth spurt such as puberty	• Increase natural protein by 0.5-1 g/day protein or phenylalanine by 25 to 50 mg/day if blood phenylalanine levels are consistently below target range. • Increase by a further 0.5 to 1 g/day protein or phenylalanine by 25 to 50 mg/day for every 3 consecutive blood phenylalanine levels below target range.
Excess intake of infant protein substitute or overnight consumption of infant protein substitute.	• Infants may take phenylalanine- reduce gap free infant protein substitute overnight, which may lower morning blood concentrations. Consider reducing overnight intake if appropriate.
No obvious reason	• Consider increasing natural protein by approx. 0.5-1 g protein or phenylalanine by 25 to 50 mg/day. • Monitor blood phenylalanine levels carefully. • It is good practice to re-check blood phenylalanine levels before any further increase in natural protein /phenylalanine intake.

Table adapted from MacDonald A, White F. Amino acid disorders. In Shaw V, editor. Clinical Paediatric Dietetics: Chichester: Wiley Blackwell; 2015. p. 433–434

## Management during illness

During illness poor appetite and low energy intake are common, and high blood phenylalanine concentrations occur due to catabolism (protein breakdown). There is little work defining the best management for illness in PKU, but the guidelines in Table 8 may be helpful. All children should complete their child health vaccination program to protect them from infectious diseases which may otherwise impact on blood phenylalanine control.

**Table 8:** Factors to consider during illness management in PKU

Diet	Dietary advice
Protein substitute	Maintenance of protein substitute intake is essential to help minimise catabolism during illness. It is better to give this in smaller, more frequent doses throughout the day. Protein substitute given when there is a high temperature may lead to vomiting. It is always better to administer protein substitute when a high temperature is brought under control post administration of anti-pyretic medication.
High carbohydrate intake	Encourage frequent high carbohydrate drinks or glucose polymer solution.
Natural protein intake	If on dietary treatment only, there is no need to ‘formally’ omit natural protein. In practice, a reduced appetite leads to a lower natural protein intake. If on sapropterin (and dietary treatment), advice is the same as for dietary treatment only.
Medications	All treatment specific medication should be continued during illness. Medications should be free of aspartame. Continue sapropterin if prescribed.
Treat precipitating factors	Antibiotics (aspartame-free if possible)^a^.
Anti-pyretic medication	Give as necessary for high temperatures.

^a^Blood phenylalanine concentrations are likely to rise quickly during illness. For the immediate and short-term treatment of infections, if only aspartame containing medicines are available, it may be better to use these until aspartame-free medication is sourced rather than leave a person with PKU without such treatment

## Infant feeding

Infants with untreated blood phenylalanine > 360 μmol/L on diagnosis are treated with a low phenylalanine diet. This involves controlling and lowering the amount of natural protein intake by giving less breast milk or standard infant formula in combination with a phenylalanine-free infant formula. Infants diagnosed > 10 days of age may have a diagnostic level in excess of > 1000 μmol/L and should be given phenylalanine- free infant formula only until blood phenylalanine levels < 1000 μmol/L are achieved. Full term infants meeting their fluid requirements with phenylalanine- free infant formula only, should be expected to lower blood phenylalanine concentrations by around 400 μmol/L each day; the number of days on a phenylalanine-free formula only should be limited to avoid phenylalanine deficiency.

### Breastfeeding

The European PKU guidelines recommend that breast feeding is encouraged in preference to using standard infant formula as a source of phenylalanine; there is considerable experience of breast-feeding infants with PKU [19–21]. Acceptable blood phenylalanine control, growth and weight gain is attained provided it is administered alongside a phenylalanine- free infant formula.

Breast milk has many advantages when compared to standard infant formula:

- it is low in phenylalanine (46 mg/100 ml) and contains long chain polyunsaturated fatty acids such as docosahexaenoic acid.
- demand breast feeding helps establish good mother-infant bonding.
- demand breast feeding gives the mother some control over the feeding process.
- demand breast feeding is convenient and reduces the number of bottle feeds required.

The most widely reported method of breast feeding is based on the principle of giving a measured volume of a phenylalanine- free infant formula before each breast feed. This reduces the infant’s appetite and hence suckling, thus lowering the amount of breast milk taken and thereby lowering phenylalanine intake [22]. Infants still feed on demand, varying the number of feeds from day to day, but the phenylalanine- free infant formula should always be given first. Blood phenylalanine concentrations determine the volume of phenylalanine- free infant formula required and the feed volume commonly varies from between 30 to 60 ml. Most breast-fed infants take from 6 to 8 feeds/daily of phenylalanine- free infant formula prior to breast feeds. It is good practice to encourage parents to keep records of daily feeds taken. Alternatively, breast feeds can be given alternately with the phenylalanine- free infant formula although there is less evidence to support this. The number of breast feeds per day is based on the blood phenylalanine results [23].

Initially, mothers need ongoing reassurance and practical support, particularly understanding their infants’ cues for feeding and managing any common breastfeeding difficulties. At first, mothers may feel their baby is taking inadequate breast milk if they are slow to recommence suckling. Blood phenylalanine concentrations should be checked twice weekly until they have stabilised. The baby requires weighing weekly for at least the first 6 weeks. Frequent weights help provide reassurance that the infant is gaining weight and by inference, taking enough breast milk. Ideally, breast feeding should continue long term.

### Bottle feeding

When standard infant formula is used as the phenylalanine source, it is given in combination with phenylalanine free infant formula. Fifty milligrams per kilogram of phenylalanine from standard infant formula is administered as a starting point, and then the dose is titrated according to blood phenylalanine levels. The total daily amount of infant formula is divided between 6 and 7 feeds. The rest of the fluid requirements is provided by phenylalanine-free infant formula.

The total volume provided by the two formulas should equate to a feed volume of 150–200 mL/kg/day. The phenylalanine-free infant formula and the source of phenylalanine (standard infant formula) should be given at the same feed to deliver the correct balance of all essential amino acids.

Traditionally it is recommended the standard infant formula is given first to ensure the entire phenylalanine source is completed, followed by the phenylalanine-free infant protein substitute, which can be fed to appetite, with guidance on an acceptable minimal volume to provide total protein requirements. Alternatively, the calculated volume of normal infant formula and phenylalanine-free infant protein substitute can be mixed. This is the most popular method in Europe [22]. A disadvantage of mixing the source of phenylalanine (standard infant formula) with phenylalanine-free infant formula is that the taste may be rejected when the amount of standard infant formula is reduced or removed. Ensuring that a minimal volume of phenylalanine-free infant formula is consumed is just as important as ensuring that the phenylalanine requirement is met but not exceeded.

The quantity of standard infant formula is adjusted by amounts equivalent to 25-50 mg/day phenylalanine according to blood phenylalanine concentrations. If blood phenylalanine concentrations are < 120 μmol/L the standard infant formula is increased. If blood phenylalanine is > 360 μmol/L, the dietary phenylalanine is decreased by 25-50 mg/day, providing the infant is well and drinking adequate quantities of phenylalanine-free infant formula.

### Weaning

Most PKU centres introduce solids from between 17 to 26 weeks of age which is a little earlier than the recommendation of 26 weeks for the general population [9]. In PKU, weaning development is similar to non PKU infants. Evans et al. 2019 showed that progression onto more textured foods, reduction in infant formula intake, and self-feeding skills were comparable between infants with PKU and non-PKU matched controls [24].

Across Europe, the most common first foods are fruit and vegetables that contain phenylalanine ≤75 mg/100 g e.g. apple, pear, carrot, butternut squash, sweet potato. Initially, these are offered once daily, and it is probably better to give low protein weaning foods after the infant phenylalanine- free infant formula and breast or standard infant formula feeds so as not to inhibit appetite for ‘milk’. Many low protein weaning foods have a low energy density so this should be increased by adding low protein milk, oil, butter, margarine, plant cream or low protein cereal. Weaning foods are gradually increased to 3 times daily. Progressively, measured amounts of phenylalanine/protein from food are introduced to replace the phenylalanine/protein from breast or formula feeds. Foods such as mashed potato, peas, yoghurt, ordinary rusks, baby rice/baby cereal or vegetable-based weaning foods in jars or tins are useful natural protein exchange foods. Foods with more texture are introduced from 6 to 8 months. Finger foods such as fingers of low protein toast, soft fruits such as bananas, strawberries and peaches, soft vegetable sticks, fingers of low protein cheese, homemade low protein bread sticks, low protein rusks and biscuits can be given from 7 months of age. Water can be given from a feeder beaker at 7 months of age.

From 9 months, low protein pasta dishes (e.g. low protein macaroni with low protein cheese sauce), sandwiches made with low protein bread and low protein cheese, chopped low protein burgers or sausages with oven baked vegetable chips, or low protein mince (made with finely chopped mushrooms) served with mashed potato and vegetables are suitable foods.

From the age of 6 months, it becomes necessary to introduce a more concentrated protein substitute or second stage protein substitute. This is common practice in Europe [9]. The amount of second stage protein substitute is gradually increased in order to meet total protein requirements. The protein substitute can be an unflavoured powdered protein substitute given in the form of a semi-solid, spoonable or gel presentation and most are suitable from the age of 6 months. These are mixed with a small quantity of water and administered 3 times daily. Alternatively, a second-stage protein substitute may be given as a drink, although the volume required may depress the appetite for food.

## Feeding behaviour in young children

Feeding problems are common in young children with PKU [25]. Principle problems include slowness to feed, a poor appetite, and a limited variety of foods consumed. Research suggests that parents’ resort to more mealtime coercive strategies than with non PKU children to persuade them to eat, they give less verbal encouragement at mealtimes and are more likely to feed their children in isolation. Many difficulties with the administration of the protein substitute have been described in young children [26].

Reasons for feeding difficulties include:

- *Energy content of protein substitutes*. Some of the common protein substitutes provide additional energy from fat and carbohydrate; providing up to 25% of the energy requirements of a child aged 1 to 3-years. Parents may have unrealistic expectations of how much food their children should eat and persuade them to eat when they are not hungry.
- *Refusal to eat phenylalanine allowance*. Parents may be very anxious about ensuring their children eat all their phenylalanine allowance, leading to excessive attention and potentially resulting in repeated food refusal. Parental force feeding may follow causing an unpleasant mealtime situation.
- L*ack of verbal encouragement at mealtimes*. Parents may prepare two meals at each family mealtime, and for pragmatic reasons this may lead to isolated mealtimes for children with PKU, having a negative effect on appetite and feeding [27].
- *Difficulty in giving the protein substitute*. Negative behaviours such as crying, screaming, gagging, vomiting and deliberately spilling the protein substitute are common in this age group.
- *Food neophobia* is common in children with PKU and they reject new and novel foods in preference for familiar ones [28, 29]. This may be associated with lack of exposure to a wide variety of foods in the weaning period, fear of eating unsuitable foods, or unpleasant associations with protein substitute.

Table 9 provides an overview of strategies to improve feeding problems. Table 10 provides helpful hints for protein substitute administration with young children.

**Table 9:** Strategies to improve feeding problems in children with PKU

Parents should eat with children to encourage positive role modelling and social interaction [30]. Eating together, with at least one suitable low protein shared dish allows children to observe their parents eat and enjoy their foods.	
Develop consistent mealtime routines, so that parents help children learn to anticipate when they will eat. The intake of sweetened drinks or low protein milk should be controlled.	
Parents should give a suitable, healthy and varied low protein diet. Offering a child too many food choices is confusing and may cause conflict and toddler tantrums.	
Parents should allocate adequate time for each meal. When mealtimes are too brief (< 10 min) children may not have enough time to eat, particularly when they are acquiring self-feeding skills. In contrast, sitting for > 20–30 min is often difficult, and mealtimes may become aversive.	
Increasing familiarity with the taste of a food increases the likelihood of acceptance. Offer new foods several times, even if initially rejected.	
Children should be encouraged to ‘play with food’ e.g. decorate low protein biscuits or garnish a low protein pizza with vegetable toppings. This will ensure their food is fun.	
Friends should be invited to low protein birthday parties, teas and picnics. Low protein food enjoyed by others, will help food acceptance.

**Table 10:** Guidance for giving protein substitute to young children

• Establish a routine – always give protein substitute at the same time each day. • Protein substitute should always be supervised by an adult. • All caregivers should use the same consistent approach. • Administer at mealtime or with a snack. • Continue to offer even when a child refuses or is unwell. Giving a child a ‘day-off’ from protein substitute will adversely affect their blood phenylalanine control and deliver a wrong message i.e. that ‘it may be okay to stop protein substitute.’ Stopping a protein substitute even for 24 h may create difficulties with its re-introduction, particularly in young children. • It is important that children understand from an early age that their protein substitute is *just as important as any medicine*. • It is good practice to check that no protein substitute is left behind in containers or pouches.	

## Disordered eating in older people with PKU

Coping with and trying to adhere to a highly restrictive dietary treatment is a huge feature of life with PKU. It is likely to cause disordered eating by affecting feeding patterns and food choices although this is an area that has received little study in adults with PKU. Disordered eating is a descriptive phrase and not a diagnosis but is used to label a range of irregular eating behaviours. Symptoms of disordered eating include feelings of guilt and shame associated with eating, preoccupation with food, frequent dieting and anxiety associated with certain foods, and a loss of control around food. People may have been bullied about their diet and avoid eating in public.

This is an area that should be monitored closely in clinics, examining food patterns and changes in nutritional status. Patients with disordered eating are at greater risk of developing clinical eating disorders and should have an early referral to psychologists and dietitians who are experts in these disorders. Mindful eating programmes may help.

## Diet during adolescence and adulthood

Following a lifelong diet is challenging. To encourage adolescents and adults to continue their dietary treatment the following should be considered:

- All the PKU team should give a consistent message about the importance of lifelong treatment and the need for continued dietary management.
- Patients should be encouraged to take the full dose of prescribed protein substitute every day. They should try alternative protein substitutes or a combination of protein substitutes if palatability is an issue. Explore any access problems to protein substitute supply.
- Promote the following dietary practices:
  - use of plant milks instead of animal milk like cow’s milk. Some coffee shops offer plant milk as an alternative to cow’s milk.
  - vegetable foods/plant foods to replace and replicate higher protein foods: e.g. jackfruit (looks like pulled pork) in curries, stews, taco/burrito fillings, or mushroom bolognese sauce. Advocate the use of vegan/protein free cheese on pizza, sandwiches, toasties, macaroni cheese. Lower protein fish substitutes (e.g. shrimps made from konjac powder) are available.
  - use of spiralised vegetables or vegetables that have been ‘riced’, or ‘spaghetti’ squash to add extra variety. Use lower protein vegetables/plant foods like sweet potato and cassava to provide bulk and aid satiety.
  - the use of homemade chunky/ vegetable soups eaten with low protein bread.
  - regular, milk-based yoghurt and ice cream should be replaced with coconut yoghurt and plant-based ice cream.
  - use of spices, seasonings and sauces to help improve the palatability and acceptance of vegetable dishes.
  - attendance at low protein cooking classes for help with preparing basic low protein dishes. Parents/grandparents have a valuable role in helping with preparation of low protein meals in all age groups.
- Provide practical resources or suggest websites which help with menu planning, practical food preparation and basic shopping lists.
- Encourage online supermarket food shopping for food. A standard shopping list can be used from week to week; it will help ensure that suitable food is always available.
- Organise home delivery of protein substitutes and low protein food (if this option is available).
- Promote regular blood spot sampling.
- Suggest appropriate lower protein vegan /gluten free options when eating out.
- At clinic visits discuss food challenges. Discuss how events like holidays, or family gatherings have been managed.
- Explore any financial barriers that may limit ability to adhere to diet and signpost the patients to appropriate advice and support.
- Ensure there is a smooth transition between paediatric and adult care with appropriate education, which will help equip and support the adolescent to sustain dietary management. Useful considerations that will assist in the transition process are given in Table 11.

**Table 11:** Useful considerations that will assist in the transition process between paediatric and adult care to help sustain dietary management

**Written healthcare transition plan** Patients and families need an individualized care plan and timetable for transition. This plan should include treatment goals, a timetable for transfer, and ensure there is a consistency of approach between all health professionals. The care plan should include information about target blood phenylalanine levels, expected frequency of home blood spot taking, educational needs and the training skills required by the patient.	
**Necessary knowledge and skills required for independent living** This should include information about the low phenylalanine diet e.g. natural protein/phenylalanine allowance, dose of protein substitute, suitable low protein foods, meal planning and food preparation, ordering of dietary products on prescription, travel/holidays and eating away from home. Patients should be encouraged to use apps to record blood phenylalanine levels, reminders for protein substitute intake and calculate their daily protein/phenylalanine intake. They also need a good understanding of the possible effects of high blood phenylalanine levels on mood, cognitive and executive function.	
**Home blood taking/blood results** Patients should learn to take home blood samples competently, order blood equipment and return samples according to agreed schedules. Teenagers should discuss their own blood results directly with health professionals. Blood results could be sent by text, telephone, or by computer web sites (providing this is permitted by hospital IT privacy policies) to teenagers.	
**Lifestyle issues** Consider overweight, body image, healthy lifestyle, exercise, extreme sports, smoking and its cessation, alcohol, recreational drugs, pregnancy, contraception, and genetics.	
**Psychological and social support** Peer support, effective strategies to cope with bullying and feelings of social isolation should be explored. Consider provision of ‘role model’ peer support, peer support groups, suitable and ‘monitored’ ‘facebook’ or internet chat sites. Information and support are required regarding changes in financial allowances, grants, insurance or prescription charges. Consider ‘supported’ summer camps for adolescents and adults to share and discuss issues that concern them.	
**Supporting the caregivers** Involve parents/caregivers at all stages. Give parents/caregivers information and listen to their concerns. They will need time to develop trust in the adult IMD team. At the start of the transition process, information should be given to parents about the philosophy of transition, so they can prepare for the change from the paediatric to the adult team.	
**Transfer timing** Consider other changes and events in a patient’s life: exams, school leaving, university commencement, and relationships. Consider developmental readiness.	
**Preparing for adult service** Teenagers should be seen in clinic without parents/caregivers but still give parents the opportunity to discuss any concerns they may have. The adult team should attend transition clinics and be introduced to the family and young adult from the age of 14 years. A pre-transfer visit to the adult clinic may be helpful with at least one return visit made to the paediatric clinic to discuss concerns. Ensure a member of the paediatric team attends the first few adult clinic visits to ensure continuity of care and a familiar face.	

Table adapted from MacDonald A, White F. Amino acid disorders. In Shaw V, editor. Clinical Paediatric Dietetics: Chichester: Wiley Blackwell; 2015. p. 451

## Weight management

Although overweight is not uncommon, patients should be encouraged to contact their PKU team before commencing any weight reducing programme. Rapid weight loss may lead to catabolism and poor blood phenylalanine control. Weight loss should be controlled, and blood phenylalanine levels should be carefully managed. Weekly weight checks should be conducted.

### Encourage the following dietary changes

- Reduce sweetened drinks and replace with sugar free/aspartame free drinks or water.
- Increase fruit and vegetables (containing phenylalanine ≤75 mg/100 g).
- Check the carbohydrate/energy content of the protein substitute and consider a lower carbohydrate preparation. Ensure that powdered preparations are prepared with water only.
- Replace special low protein milks containing ≥60 kcal/100 ml with lower energy plant milks.
- Eat smaller portions at mealtimes but still encourage 3 meals/day using low protein pasta, rice and a low protein bread with a lower oil content.
- Limit sugar, sweets, crisps, vegetable chips, jams, honey, low protein chocolate or biscuits.
- Use minimal oil (preferably olive oil) or ‘light’ oil cooking sprays.
- Encourage 30 to 45 min physical activity each day (and at least 300 min per week).

## Sport and nutrition

As there is little information about the impact of a low phenylalanine diet affecting athletic/sporting performance, general sports nutritional requirements are modified for PKU.

The main objectives of sports nutrition and adapted by Rocha et al., 2019 [31] for PKU are:

- Maintain a high carbohydrate diet. Carbohydrate-rich foods should be recommended pre and post exercise and should include low fat, low-protein foods e.g. low protein pasta. Sports drinks (aspartame-free only) contribute to carbohydrate loading. The target carbohydrate intake in endurance exercise of 1–2.5-h duration is 30–60 g/h [31].
- Careful attention to hydration status. Monitor weight pre and post exercise which will give an indication of the fluid that should be replaced.
- Give a dose of protein substitute intake in the immediate post-exercise recovery phase. Approximately 20–30 g of protein equivalent from protein substitute should be ingested post exercise.

There is evidence to suggest that short acute exercise does not affect blood phenylalanine levels but the impact of endurance exercise has not been examined [32].

## Pregnancy

In maternal PKU, high blood phenylalanine levels during pregnancy have a teratogenic effect on the developing fetus, which can result in fetal intrauterine growth retardation, infant low birth weight, facial dysmorphism, microcephaly, developmental delay, intellectual disabilities, and congenital heart disease.

### Promoting a planned pregnancy

The European PKU guidelines (2017) state that *from the age of 12 years (beginning of puberty); all women should receive systematic age-related sex education, with professional counselling about the risk of unprotected sexual contacts. They should be informed that unplanned pregnancy can occur even during the first menstrual cycle.* Therefore, women need additional professional support throughout their reproductive life [1].

### Planning a pregnancy

Women with PKU should optimize metabolic control pre-conception and during, pregnancy. The following actions are advised:

- Meet with the PKU team 4 to 6 months prior to their anticipated start of pregnancy so they can provide guidance on achieving and maintaining safe blood phenylalanine levels prior to conception.
- Aim for blood phenylalanine levels between 120 and 360 μmol/L. If blood phenylalanine levels are higher, there should be a gradual reduction and titration of dietary phenylalanine intake until blood phenylalanine levels are within target range.
- Re-education of women and assessment of their understanding about their diet is necessary even if they already self-manage a low phenylalanine diet.
- If not established on dietary treatment, offer a variety of protein substitutes (preferably with added vitamins and minerals), until they find one that is acceptable. They need to be fully aware of all the special low protein foods available, how to access them and any cost implications.
- Give practical advice, education and support about menu planning, cooking and shopping, help with specialist food orders, and weekly food shopping lists (which could include online shopping options). This can be through practical exercises working with dietitians or diet assistants, short video’s, written and pictorial information or online information and courses.
- Teach their partner or extended family (with consent) about the diet with information about low protein food choices and cooking. Women with support are more likely to cope better with a low phenylalanine diet during pregnancy.
- Do blood spot monitoring for blood phenylalanine at least weekly in the pre-conception period.
- Maintain blood phenylalanine in the range of 120 to 360 μmol/L prior to conception for a minimum of 2 weeks or for 3 consecutive blood phenylalanine levels to demonstrate stability of blood phenylalanine control. Contraception use should continue until stability of blood phenylalanine control is maintained.
- Assess the blood spot technique. Re-education of blood spot taking technique may be necessary.
- Introduce the protein substitute if women have hyperphenylalanineamia or they have been off diet for many years. They may have no dietary treatment experience, or this may have been in the distant past. It may be necessary to introduce the full dose of protein substitute gradually over several days or weeks.
- Start folic acid supplements.

The PKU team should:

- Provide all women with contact information for the PKU team.
- Go through a check list of all necessary actions. A list of necessary pre-conception activities is given in Table 12.

**Table 12:** Check list for pre-conception diet

**•** Document height/weight/BMI **•** Perform baseline nutritional biochemistry **•** 3-day diet history to establish protein and nutritional intake /dietary knowledge /history of diet therapy /cooking skills **•** Blood phenylalanine control history **•** Establish a woman’s work patterns/shift patterns and home support **•** Awareness of any co-morbidities/other medications **•** Dietary education **•** Organise dietary supplies **•** Try different protein substitutes, if appropriate **•** Start strict dietary treatment **•** Start folic acid **•** Patient given clinic contact /helpline numbers **•** Patient should be advised to continue contraception until satisfactory blood phenylalanine is achieved.	

*Abbreviations*: *BMI* Body Mass Index

### Unplanned pregnancy

- If women do become pregnant unexpectedly or suspect they are pregnant, they should know that they must contact their PKU clinic immediately. Women should be given an emergency card with a guide on the action they should take with contact details of their PKU clinic (Table 13).
- Patients should be given an emergency box consisting of a 7-day supply of protein substitute, essential low protein special foods (e. g. low protein bread, pasta, flour and crackers), blood phenylalanine monitoring equipment, low phenylalanine diet information (including their phenylalanine allowance and dose of protein substitute), low protein recipes and important contact details for the PKU team and other services. Women may need further supplies of protein substitute or special low protein foods until their own supplies can be accessed through their health or insurance systems.
- Baseline blood phenylalanine concentrations, anthropometry, and nutritional biochemistry should be established.
- Women will need to start dietary treatment immediately. Stop all high protein foods and commence phenylalanine restriction. As a starting point, the initial phenylalanine allocation should be the same as the phenylalanine tolerance when aged < 5 years of age [33].
- Women will need to start their full requirement of protein substitute from the day of starting dietary treatment. It is recommended that women take ≥70 g/day total protein (calculating both natural and amino acid intake from protein substitute). Protein requirements have not been assessed in PKU pregnancy and can only be extrapolated from non PKU pregnancy. Most women will need to start on at least 60 g/day protein equivalent from a protein substitute, but the dose will need to be individually calculated by the hospital team.
- Women should be given a 7-day individualized menu plan, with a shopping list and recipe cards to give them a guide as to what they should eat whilst they adjust to their diet and pregnancy. Ideally a home visiting support worker or availability of online support could help women through the early days of pregnancy.

**Table 13:** Suggested emergency card for pregnancy

**EMERGENCY PREGNANCY CARD** If you think you may be pregnant, contact your PKU clinic **immediately** Contact name: Tel Email **THE PKU TEAM WILL ORGANIZE TO SEE YOU WITHOUT DELAY.** Please take the following action • If you have supplies of protein substitute, take your full daily amount each day. • Reduce your phenylalanine intake and speak to your PKU team. They will give further advice. • Take blood spots for blood phenylalanine and post to the hospital laboratory.	

### Controlling blood phenylalanine during pregnancy

Women should be encouraged to:

- Take protein substitute as prescribed and at set times each day, at least 3 to 4 times daily.
- Eat regular small, frequent low phenylalanine meals. Low protein special foods are an important source of calories and should be incorporated into the diet each day. Weight loss can lead to higher blood phenylalanine levels.
- Maintain frequent blood spot monitoring at least twice weekly. Provide a calendar when to take blood phenylalanine samples.
- Maintain phenylalanine allowance as prescribed. Keep a record of daily phenylalanine intake. Maintain regular communication between patient and dietitian.
- Blood phenylalanine levels should be reported to women immediately when analysed by the hospital laboratory.

### Adjusting phenylalanine intake during pregnancy

- It is well established that from 16 to 20 weeks’ gestation, phenylalanine tolerance is likely to increase as foetal growth accelerates, providing women are taking their protein substitute and low phenylalanine foods as prescribed. Therefore, as the baby grows it is necessary to give extra phenylalanine to prevent phenylalanine levels below the lower limit of the target range. Persistent low phenylalanine levels, below 100 μmol/L, have been associated with intrauterine growth retardation.
- Increase dietary phenylalanine prescription by at least 50 mg/day (1 g/day protein) if blood phenylalanine levels are around or below 120 μmol/L.
- Some women find increasing phenylalanine allowance difficult, particularly if they have been on a strict low phenylalanine diet all their life. They will need guidance on when they can use higher protein foods to make up their phenylalanine allowance. For protein tolerance up to 10 g/day (500 mg/day phenylalanine), it is appropriate to use foods such as yoghurt, cow’s milk, cream cheese, breakfast cereals, rice, peas, spinach, broccoli, potatoes to make up protein allowance. When the phenylalanine allowance exceeds 500 mg (>10 g natural protein)/day, introduce protein sources such as regular bread, pasta, chick peas, cous-cous, lentils, quinoa, Quorn; and over 1000 mg/day phenylalanine (>20 g protein), high protein foods such as well-cooked egg, cheese, soya, and tofu may be necessary. Women should be given meal and snack ideas listing their phenylalanine and protein content.
- At the end of pregnancy, due to hormonal causes or a change in fetal growth, phenylalanine concentrations may increase gently at a stable phenylalanine intake, usually not needing intervention.

### Common difficulties during pregnancy

During pregnancy, similar difficulties to non PKU pregnancies may occur, but their consequences may be more important. Many women feel nauseous during pregnancy, especially in the first trimester, causing difficulties in protein substitute tolerance. Heartburn and constipation may also be a problem.

Safe anti-emetic therapy and acid reducing medications should be considered with persistent vomiting and symptoms of dyspepsia and indigestion. Advice from the family physician or obstetrician should be sought early. Useful strategies for persistent nausea, vomiting, dyspepsia and indigestion are given in Appendix 3.

Nausea and vomiting associated with pregnancy usually lessens after 12–16 weeks gestation, but symptoms of indigestion, heartburn, and constipation are more common after week 20 of pregnancy.

### Nutritional supplements during pregnancy

Requirements of vitamins and minerals should be met by the protein substitutes (providing the full dose is adhered to) but there are some exceptions.

- All women with PKU should be given a folic acid supplement that provides 400 μg/day from the time of starting a pre-conception diet until 12 weeks of pregnancy.
- There are no recommendations to give additional tyrosine during pregnancy providing women are taking their full requirement of protein substitute.
- Docosahexaenoic acid supplementation of 200 to 300 mg/day should be given to all pregnant women with PKU. This is likely to be already supplied by the protein substitute. If the protein substitute does not contain any docosahexaenoic acid, separate over-the counter fish oil supplements should be given.

### Weight gain in pregnancy

Overall, in maternal PKU weight gain should be similar to the healthy population with emphasis on avoiding weight loss particularly in the first trimester of pregnancy (Appendix 4).

- Energy requirements vary considerably for individuals but low energy intake, accompanied by weight loss is common, particularly in the first trimester of pregnancy and this is associated with higher blood phenylalanine concentrations.
- Inadequate energy intake may be due to dislike of low protein foods, poor adherence with protein substitute, limited availability of low protein foods, inability to prepare low protein meals or poor appetite associated with nausea and vomiting.
- If there is maternal weight loss, additional energy from low protein foods (e.g. pasta, bread, pizza, low protein cheese, fats, oils, low protein cream, jam, high calorie ‘milk’ shakes or hot chocolate using low protein milk) should be encouraged. Regular snacks including low protein savoury or sweet muffins, waffles, pancakes with added maple syrup /puree fruit are also encouraged. It may be necessary to use energy supplements such as glucose polymer/fat emulsions. Consider using a protein substitute with a higher energy content.
- Weight should be monitored weekly until weight gain is satisfactory.

### Obesity and maternal PKU

Any women with obesity should be encouraged to undertake more exercise and follow a calorie restricted diet prior to pregnancy consideration. The risks imposed by excessive adiposity will be cumulative to those resulting from PKU itself.

### Post pregnancy

Many women stop their dietary treatment following the birth of their baby. They may not perceive a need for continued treatment, or they may consider the diet too challenging when caring for their infant. However, health professionals should support the continuation of dietary treatment. Women should understand that high blood phenylalanine levels are associated with a higher rate of depression. Maternal depression is associated with lower rates of breast feeding, less sensitive parenting style, and poor infant sleep routines. They may cope better with the demands of parenting if blood phenylalanine levels are lower.

Women should be encouraged to breast feed their infants. Women with PKU (on or off dietary treatment) can still breast feed their children and lactation is considered successful if their infant is gaining an appropriate amount of weight. Even if a woman with PKU has a PKU infant she is still able to breast feed providing breast feeding given in combination with with a phenylalanine-free-free infant formula (see section on infant feeding). There is anecdotal evidence (but no published information) to support the hypothesis that breast feeding may help to lower blood phenylalanine levels post pregnancy. It is also likely to help maternal weight loss (at the rate of 0.6 to 0.8 kg per month in the first 4 to 6 months of lactation). Lactation does increase energy (by 500 kcal/day) and protein (25 g/day) requirements. A woman’s weight, BMI, body fat percentage, and weight gain during pregnancy do not influence breast milk production [34].

Women need support during breast feeding, they should be encouraged to take their protein substitute at least 3 times daily as well as eating regular low protein meals and snacks during the day.

Ideally, women should be encouraged to ‘batch cook’ and freeze low protein dishes, stock cupboards with low protein basic foods whilst they are still pregnant are organised and prepared for the postpartum period. They could arrange supermarket home deliveries with regular supplies of fruit and vegetables. Use of frozen chopped fruit and vegetables will save on preparation time.

Women also need to be aware that the postpartum period is a high-risk time for unintended pregnancy, so family planning and contraceptive advice remains important.

### Use of sapropterin in pregnancy

Women should be tested prior to pregnancy (either by genotyping or a BH4 loading test) [35]. If women fail to establish metabolic control in pregnancy, in sapropterin responsive women this drug should help lower blood phenylalanine levels and reduce the requirement for stringent diet therapy, especially, in the first 12 weeks of pregnancy. A higher natural protein intake is associated with better fetal growth [36].

## Support and information resources

Patient education lies at the core of managing a low phenylalanine diet. Effective low protein education is fundamental in bringing about a change in eating behaviour and family lifestyle. Education should foster lifelong self-care that will ensure optimal metabolic control and social outcomes (Table 14).

**Table 14:** Educational methods used in PKU

Teaching methods	Recommendations
**Verbal/face to face** Most common and effective method.	• Spend time with patients and caregivers and explain the diet ‘one to one,’ answering immediate questions. Try to make sessions very practical using aids such as food package labelling, web-based supermarket shops to plan meals, and demonstrate how to make up protein substitutes. • The end of every ‘verbal’ teaching session should include 'feed back time’ whereby patients/caregivers’ feedback what they have learnt. •. Patients/caregivers should be given a short, written summary (e.g. 4 to 5 action points) about advice given. • Brief follow-up telephone calls (e.g. reiterating dietary principles) are effective and increases adherence. • Offer face to face teaching to grandparents, family caregivers, nursery and school teachers.
**Written materials/apps** Written materials are commonly used to reinforce verbal education given to patients and families.	• Written information sheets should be available for caregivers, children, adolescent and adult patients with PKU, and others involved in management. It is also important that written materials are available about diet and non-diet treatments. • Dietitians should involve caregivers and patients in developing materials. • All new written information should be pilot tested with a group of patients/ caregivers to assess its ‘usability’ factor. • Provide written educational packages for nurseries and schools. • Apps that provide basic information about diet, phenylalanine content of foods, phenylalanine counting, reminders to take protein substitute and blood phenylalanine tracking are helpful. However, apps are expensive to produce, and many are produced by commercial companies so may not be independent in the information they provide. It is important to check patient aids before recommending to patients.
**Pictures** Adding pictures to written and spoken language can increase patient attention, comprehension, recall and adherence	• Pictures are useful to show step by step procedures for administering protein substitutes, preparing recipes, and blood sample taking.
**Children’s group teaching** Group practical sessions encourage self-management and help develop social support networks.	• Practical activities should be provided. Children may plan meals, shop for food, weigh and measure foods, and prepare meals and snacks. • All activities should be interactive, creative, and fun. • Attendance at a ‘PKU school/forum’ could replace a routine clinic visit or run parallel to a clinic. • Certificates and learning credits should be issued to encourage attendance. • If patients are scattered in large geographical areas, the internet and other multi-media approaches could be used for ‘group’ teaching.
**Peer support and patient advocates** These are caregivers or patients who can give practical help and support.	• A PKU home support worker with personal experience of PKU care and working one to one with families can help others build confidence, improve cooking skills, diet knowledge and overall parenting skills. • Encouraging caregivers to network with others is good for sharing experiences, discussing coping strategies, and sharing practical information. This is particularly important for ‘new parents’ of children with PKU. This can be done through local and national events or ‘Facebook’, Instagram or other similar forums. • Working with peer ‘patient ambassadors’ may act as a motivator and guide. • Women who have experienced pregnancy with PKU can act as role models to teach and support girls and women with PKU.
**Web-based patient education and support** Patients/caregivers can work through teaching modules at their own pace, and gain immediate feedback from interactive programmes	• National and international teaching PKU packages for different ages of patients should be developed, with computer marked assessment of knowledge learnt, with feedback to the PKU health professional team. • For adolescents and adults with PKU, signing up to web-based ‘mindfulness’ programs to help well-being may be useful. • European websites such as the ESPKU contain information about travelling with PKU, information about the phenylalanine content of common European foods, explain European food law and implications for food labelling. • National societies should provide more standard resources e.g. ‘print off’ letters to give to schools, nurseries, airport security, hotels, restaurants explaining about PKU and a low phenylalanine diet. • ‘You Tube’ educational videos are useful to explain about diet to all ages and intellectual abilities.
**Re-evaluation and audit of knowledge**	• It is important to re-evaluate caregiver/patient knowledge, understanding, and interpretation of dietary principles once every 2 to 3 years. Even an update providing new information is valuable.

### Useful practical resources

#### Scales

A good set of food digital weighing scales is important for measuring protein/phenylalanine portion sizes or protein substitute powder accurately. It is essential that every patient/caregiver with PKU ‘owns’ a reliable set of food scales; it is an important investment.

Important scale characteristics are:

- has a tare feature that subtracts the weight of the weighing container, so it provides the weight of the ingredients only.
- numbers are clear, large and easy to read.
- it is lightweight and preferably portable.
- weighs in 1 g units.

#### Useful cooking equipment

a bread, soup, waffle and pancake maker, slow cooker and food processer help save time and aid variety.

#### Wall organization boards

A white board that will record either 7-day menu plans, shopping lists, date reminders for ordering of protein substitutes, specialist low protein foods or hospital appointments or recording of phenylalanine intake are invaluable.

#### Protein exchange calculators

**Fig. 2:**
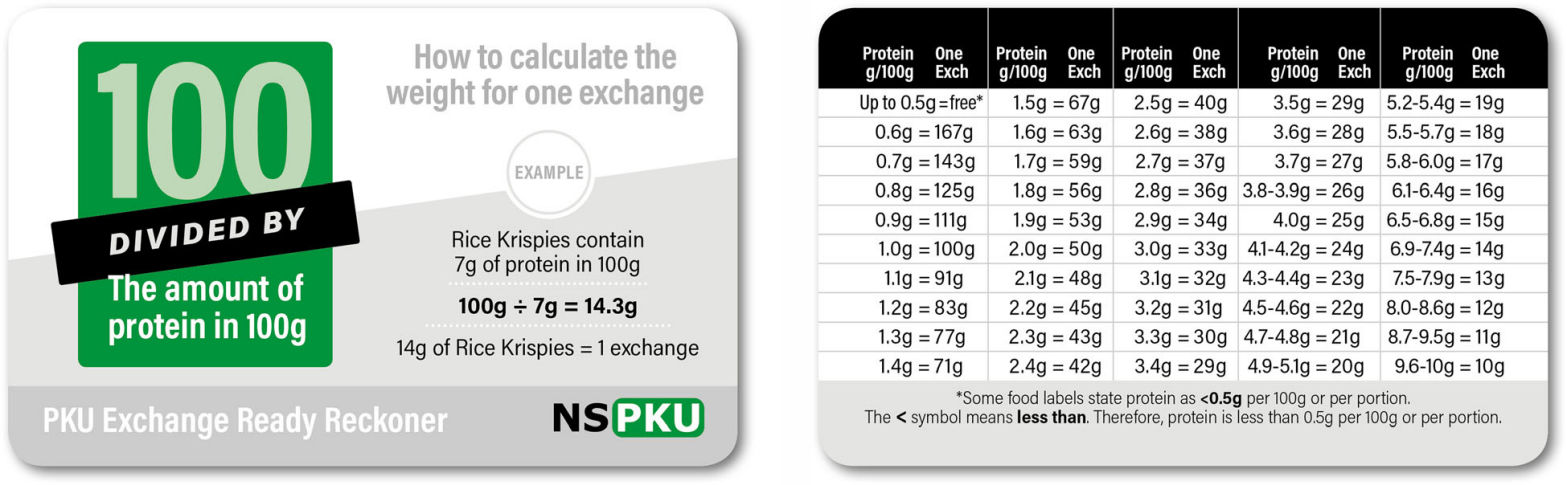
Protein exchange calculator (NSPKU website 2019, download)

This may be an app or card that has been formulated to help patients/parents to calculate the amount of food that will provide 1 g protein or 50 mg phenylalanine from food labels (Fig. 2).

## Alcohol

Like the rest of the population, adults with PKU are advised to take alcohol in moderation.

However, there are many suitable alcoholic drinks for people with PKU (Table 15). Some alcoholic drinks do contain protein such as beer and should only be taken as part of the daily protein/phenylalanine allowance. Unfortunately, there are no European food laws that mandate that nutritional information is provided on alcoholic drinks. This means that alcohol may not list the protein content per 100 ml on food labelling unless a manufacturer chooses to declare this voluntarily.

Also, some drinks contain aspartame and should be avoided.

**Table 15:** Suitable alcoholic drinks

- Dry, sweet and vintage cidar. - Red, white and rose wine (750 ml contains approximately 50 mg phenylalanine). - Champagne and prosecco. - Port wine and sherry. - Dry and sweet vermouth. - Spirits: whisky, gin, vodka, rum, brandy, tequila and martini. Please note pre-mixed drinks and cocktails (e.g. alcohol + lemonade may contain aspartame). Alcoholic ‘creams’ and some liqueur’s such as Amaretto and Advocaat (containing egg) will contain protein. **Beer contains a significant amount of phenylalanine. Generally, the stronger the beer the higher the protein content, because it usually contains a higher malted cereal content. 600 ml beer will vary between 75 to 100 mg phenylalanine.**	

## Conclusions

This practical resource should help health professionals deliver PKU dietary management according to the statements issued by the PKU European Guidelines [1]. Dietary management, with or without sapropterin, is complex and requires diligent attention to detail. Generally, across Europe there are still many countries with limited numbers of dietitians trained in PKU management, and in some countries, it is the patient support groups who provide considerable practical support and are dependent on evidence-based resources. The PKU Guidelines group not only considers it their responsibility to develop recommendations for management but also to support the PKU community with the provision of practical information to help with guideline implementation.

## Definitions

**PHENYLKETONURIA,** also known as PKU, is an inborn error of metabolism due to phenylalanine hydroxylase deficiency. It causes decreased metabolism of an amino acid called phenylalanine. Untreated PKU causes intellectual disability, seizures, and behavioural problems.

***PHENYLALANINE*** is an essential amino acid found in food. Major dietary sources of L-phenylalanine include meat, fish, eggs, cheese, and milk. It is a precursor for tyrosine, and subsequently the neurotransmitter dopamine and the skin pigment melanin.

**PROTEIN SUBSTITUTE** is also referred to as medical food or synthetic protein. It is a protein usually made up of essential, semi-essential and non-essential amino acids, without phenylalanine. Its role is to replace natural protein.

**NATURAL PROTEIN** is also referred to as intact protein. This is protein from food sources which is made of polymer chains consisting of amino acids linked together by peptide bonds.

**LOW PROTEIN SPECIAL FOODS** are “dietary foods for special medical purposes” and are defined in EC Regulations as meaning ‘*a category of foods for particular nutritional uses specifically processed or formulated and intended for the dietary management of patients and to be used under medical supervision’.* They have a low protein and phenylalanine content and are products such as flour, bread or pasta that are produced for the treatment in bold of PKU.

***SAPROPTERIN dihydrochloride***, referred to as *sapropterin*, is a synthetic formulation of the active 6R-isomer of tetrahydrobiopterin, a naturally occurring cofactor for phenylalanine hydroxylase. Sapropterin is an oral licensed drug for the treatment of PKU.

## Supplementary information

**Additional file 1.**

**Additional file 2.**

**Additional file 3.**

**Additional file 4.**

## Data Availability

Not applicable.

## Abbreviations

CGMP: Casein glycomacropeptide
FAO: Food and Agriculture Organization of the United Nations
PKU: Phenylketonuria
WHO: World Health Organization
UNU: United Nations University
