# Correction to: PKU dietary handbook to accompany PKU guidelines

**DOI:** 10.1186/s13023-020-01486-6

**Published:** 2020-09-01

**Authors:** A. MacDonald, A. M. J. van Wegberg, K. Ahring, S. Beblo, A. Bélanger-Quintana, A. Burlina, J. Campistol, T. Coşkun, F. Feillet, M. Giżewska, S. C. Huijbregts, V. Leuzzi, F. Maillot, A. C. Muntau, J. C. Rocha, C. Romani, F. Trefz, F. J. van Spronsen

**Affiliations:** 1 Dietetic Department, Birmingham Children’s Hospital, Birmingham, UK; 2 Division of Metabolic Diseases, Beatrix Children’s Hospital, University Medical Centre Groningen, University of Groningen, Hanzeplein 1, 9700 RB Groningen, The Netherlands; 3 Department of PKU, Kennedy Centre, Glostrup, Denmark; 4 Department of Women and Child Health, Center for Pediatric Research Leipzig, Hospital for Children and Adolescents, University Hospitals, Leipzig, Germany; 5 Department of Paediatrics, Hospital Ramon y Cajal Madrid, Metabolic Diseases Unit, Madrid, Spain; 6 Division of Inherited Metabolic Diseases, Department of Paediatrics, University Hospital of Padova, Padova, Italy; 7 Neuropaediatrics Department, Hospital Sant Joan de Déu, Universitat de Barcelona, Barcelona, Spain; 8 Hacettepe University Faculty of Medicine, Ankara, Turkey; 9 Department of Paediatrics, Hôpital d’Enfants Brabois, CHU Nancy, Vandoeuvre les Nancy, France; 10 Department of Paediatrics, Endocrinology, Diabetology, Metabolic Diseases and Cardiology of the Developmental Age, Pomeranian Medical University, Szczecin, Poland; 11 Department of Clinical Child and Adolescent Studies-Neurodevelopmental Disorders, Faculty of Social Sciences, Leiden University, Leiden, The Netherlands; 12 Department of Paediatrics, Child Neurology and Psychiatry, Sapienza University of Rome, Via dei Sabelli 108, 00185 Rome, Italy; 13 CHRU de Tours, Université François Rabelais, INSERM U1069, Tours, France; 14 University Children’s Hospital, University Medical Centre Hamburg-Eppendorf, 20246 Hamburg, Germany; 15 Nutrition & Metabolism, NOVA Medical School, Faculdade de Ciências Médicas, Universidade Nova de Lisboa, Lisbon, Portugal. Centre for Health Technology and Services Research (CINTESIS), Porto, Portugal; 16 School of Life and Health Sciences, Aston University, Birmingham, UK; 17 Department of Paediatrics, University of Heidelberg, Heidelberg, Germany

**Correction to: Orphanet J Rare Dis 15, 171 (2020)**

**https://doi.org/10.1186/s13023-020-01391-y**

The original article [1] contained an error in the **Sport and Nutrition** section of the manuscript whereby it was stated that:

*‘Approximately 20–30 g of protein equivalent from protein substitute should be ingested post exercise.’*

This sentence should instead have stated ‘ *… 20g of protein equivalent from protein substitute* … ’ which has since been implemented in the original article.
