# Supplementary material for: PKU dietary handbook to accompany PKU guidelines
Source: Orphanet J Rare Dis. 2020 Jun 30;15:171. doi: 10.1186/s13023-020-01391-y (PMC7329487; doi:10.1186/s13023-020-01391-y)
Supplement: Supplementary file 1 — Additional file 1. [file 13023_2020_1391_MOESM1_ESM.docx]

# Appendix 1: Systems for allocating phenylalanine in the diet

**System 1 for allocating phenylalanine in the diet**

**Meal plan using 50 mg phenylalanine exchanges**

**50 mg phenylalanine vegetable exchanges**

**15g corn based cereal**

**80g boiled potato**

**25g spinach**

**25g peas**

**Menu plan for a 4-year-old girl, with PKU on 4 x 50 mg phenylalanine exchanges daily (200 mg/day phenylalanine) and protein substitute 3 times daily**

| **Breakfast**  Phe-free L-amino acid supplement (1/3 of full dose)  1 x 50mg phenylalanine exchange corn based breakfast cereal (15g)  Protein-free milk replacement  Low protein toast (exchange-free), butter (exchange-free), fresh tomatoes (exchange-free)  Fruit juice – aspartame free  **Midday**  Phe-free L-amino acid supplement (1/3 of full dose)  Home-made vegetable soup (made from exchange-free vegetables) + add 1 x 50mg phenylalanine exchange of potato (80g)  Low protein bread - exchange-free  1 apple/melon slices - exchange-free  Water  **Mid-afternoon**  Low protein muffin - exchange-free  Water  **Evening Meal**  Phe-free L-amino acid supplement (1/3 of full dose)  1 x 50mg phenylalanine exchange of spinach (25g)  1 x 50mg phenylalanine exchange of peas (25g)  Low protein risotto (made from low protein rice and exchange-free ingredients)  Salad vegetables – exchange-free  Low protein bread + butter – exchange-free  Low protein (gelatin free/aspartame free) jelly – exchange-free  Water  **Bedtime**  Protein-free milk replacement made into ‘hot drink’ – exchange-free  Total 200 mg phenylalanine |
| --- |

**System 2 for allocating phenylalanine in the diet**

**1g protein exchanges**

**15g corn based cereal**

**55g boiled potato**

**45g spinach**

**15g peas**

**Meal plan using 1g protein exchanges**

**Menu plan for a 4-year-old girl, with PKU on 4g protein daily (200 mg/day phenylalanine) and protein substitute 3 times daily**

| **Breakfast**  Phe-free L-amino acid supplement (1/3 of full dose)  1 x 1g protein exchange of corn based breakfast cereal (15g)  Protein-free milk replacement  Low protein toast (exchange-free), butter (exchange-free), fresh tomatoes (exchange-free)  Fruit juice – aspartame free  **Midday**  Phe-free L-amino acid supplement (1/3 of full dose)  Home-made vegetable soup (made from exchange-free vegetables) + add 1g protein exchange of potato (55g)  Low protein bread - exchange-free  1 apple/melon slices - exchange-free  Water  **Mid-afternoon**  Low protein muffin - exchange-free  Water  **Evening Meal**  Phe-free L-amino acid supplement (1/3 of full dose)  1g protein exchange of spinach (45g)  1g protein exchange of peas (15g)  Low protein risotto (made from low protein rice and exchange-free ingredients)  Salad vegetables – exchange-free  Low protein bread + butter – exchange-free  Low protein (gelatin free/aspartame free) jelly – exchange-free  Water  **Bedtime**  Protein-free milk replacement made into ‘hot drink’ – exchange-free  Total 4g protein |
| --- |

**System 3 for allocating phenylalanine in the diet**

**Meal plan using 1g protein exchanges but using the phenylalanine analysis of fruit and vegetables and calculating the weight that provides 50 mg phenylalanine**

**Menu plan for a 4-year-old girl, with PKU on 4g protein daily (200 mg/day phenylalanine) and protein substitute 3 times daily**

**1g protein exchanges**

**15g corn based cereal**

**50 mg phenylalanine vegetable exchanges**

**80g boiled potato**

**25g spinach**

**25g peas**

| **Breakfast**  Phe-free L-amino acid supplement (1/3 of full dose)  1g protein exchange of corn based breakfast cereal (15g)  Protein-free milk replacement  Low protein toast (exchange-free), butter (exchange-free), fresh tomatoes (exchange-free)  Fruit juice – aspartame free  **Midday**  Phe-free L-amino acid supplement (1/3 of full dose)  Home-made vegetable soup (made from exchange free vegetables) + add 1 x 50mg phenylalanine exchange of potato (80g)  Low protein bread – exchange-free  1 apple/melon slices- exchange-free  Water  **Mid-afternoon**  Low protein muffin- exchange-free  Water  **Evening Meal**  Phe free L-amino acid supplement (1/3 of full dose)  1 x 50mg phenylalanine exchange of spinach (25g)  1 x 50mg phenylalanine exchange of peas (25g)  Low protein risotto (made from low protein rice and exchange-free ingredients)  Salad vegetables – exchange-free  Low protein bread + butter - exchange-free  Low protein (gelatin free/aspartame free) jelly – exchange-free  Water  Bedtime  Protein-free milk replacement made into ‘hot drink’ – exchange-free  Total 1g protein from food + 150 mg phenylalanine from vegetables |
| --- |

**System 4 for allocating phenylalanine in the diet**

**Paper lists/apps listing the phenylalanine content of foods**

**Phenylalanine content mg/100g**

**Corn based cereal: 430 mg**

**Potato – boiled: 60 mg**

**Spinach cooked: 193 mg**

**Peas cooked: 194 mg**

**Menu plan for a 4-year-old girl, with PKU on 200 mg/day phenylalanine and protein substitute 3 times daily**

| **Breakfast**  Phe-free L-amino acid supplement (1/3 of full dose)  15g corn based breakfast cereal (65mg phenylalanine)  Protein-free milk replacement  Low protein toast (low phenylalanine), butter (low phenylalanine), fresh tomatoes (low phenylalanine)  Fruit juice – aspartame free  **Midday**  Phe-free L-amino acid supplement (1/3 of full dose)  Home-made vegetable soup (made from low phenylalanine vegetables) + add 80g potato (48mg phenylalanine)  Low protein bread – low phenylalanine  1 apple/melon slices- low phenylalanine  Water  **Mid-afternoon**  Low protein muffin- low phenylalanine  Water  **Evening Meal**  Phe free L-amino acid supplement (1/3 of full dose)  25g spinach (48mg phenylalanine)  25g peas (48mg phenylalanine)  Low protein risotto (made from low protein rice and low phenylalanine ingredients)  Salad vegetables – exchange-free  Low protein bread + butter - exchange-free  Low protein (gelatin free/aspartame free) jelly - low phenylalanine  Water  **Bedtime**  Protein-free milk replacement made into ‘hot drink’ – low phenylalanine  Total 209mg phenylalanine/day |
| --- |

**System 5 for allocating phenylalanine in the diet**

**Paper lists/apps listing the protein content of foods**

**Protein content g/100g**

**Corn based cereal: 7.1g/100g**

**Potato – cooked: 1.8g/100g**

**Spinach cooked: 2.2g/100g**

**Peas cooked: 5.7g/100g**

**Menu plan for a 4-year-old girl, with PKU on 4g protein day and protein substitute 3 times daily**

| **Breakfast**  Phe free L-amino acid supplement (1/3 of full dose)  15g corn based breakfast cereal (1.1g protein)  Protein-free milk replacement  Low protein toast (low protein), butter (low protein), fresh tomatoes (low protein)  Fruit juice – aspartame free  Midday  Phe free L-amino acid supplement (1/3 of full dose)  Home-made vegetable soup (made from exchange free vegetables) + add 55g potato (1g protein)  Low protein bread - low protein  1 apple/melon slices - low protein  Water  **Mid-afternoon**  Low protein muffin - low protein  Water  **Evening Meal**  Phe-free L-amino acid supplement (1/3 of full dose)  45g spinach (1g protein)  18g peas (1g protein)  Low protein risotto (made from low protein rice and low protein ingredients)  Salad vegetables – low protein  Low protein bread + butter - low protein  Low protein (gelatin free/aspartame free) jelly - low protein  Water  **Bedtime**  Protein-free milk replacement made into ‘hot drink’ – low protein  Total 4.1g/day protein |
| --- |
