# Supplementary material for: PKU dietary handbook to accompany PKU guidelines
Source: Orphanet J Rare Dis. 2020 Jun 30;15:171. doi: 10.1186/s13023-020-01391-y (PMC7329487; doi:10.1186/s13023-020-01391-y)
Supplement: Supplementary file 2 — Additional file 2. [file 13023_2020_1391_MOESM2_ESM.docx]

**Appendix 2: Phenylalanine content of aspartame containing foods**

**Adapted from maximum permitted limits of aspartame (E951) in foods according to the EU Commission Regulation 1129/2011 (adapted from EFSA 2013^1^**

| **Foods** | **Restrictions/exceptions** | **Maximum level (mg/L or mg/kg as appropriate) of aspartame** | **56% converted to Phe (maximum level (mg/L or mg/kg as appropriate)** | **Estimated maximum amount of Phe per average portion if aspartame added to recipe** |
| --- | --- | --- | --- | --- |
| Edible ices | Only energy reduced or with no added sugar | 800 | 448 | 134 mg/30g portion |
| Jam, jellies and marmalades as defined by Directive 2001/113/EEC | Only energy reduced jams, jellies and marmalades | 1000 | 560 | 56 mg/100g jelly portion  11 mg/20g portion jam/marmalade |
| Confectionary including breath refreshing micro sweets | Only starch based confectionary, energy reduced or with no added sugar | 2000 | 1120 |  |
| Confectionary including breath refreshing micro sweets | Only confectionary with no added sugar | 1000 | 560 | 6 mg/per sweet |
| Confectionary including breath refreshing micro sweets | Only breath -freshening micro-sweets with no added sugar | 6000 | 3360 | 6 mg/per sweet |
| Confectionary including breath refreshing micro sweets | Only strongly flavoured freshening throat pastilles with no added sugar | 2000 | 1120 | 6 mg/per sweet |
| Chewing gum | Only with added sugars or polyols as flavour enhancer | 2500 | 1400 | 3 mg/2 g piece |
| Chewing gum | Only with no added sugar | 2000 | 1120 | 2 mg/2 g piece |
| Breakfast cereals | Only breakfast cereals with a fibre content of more than 15% and containing at least 20% bran, energy reduced or with no added sugar | 1000 | 560 | 17 mg/30g portion |
| Soups and broths | Only energy reduced soups | 110 | 62 | 12 mg/200 ml |
| Sauces |  | 350 | 196 | 4 mg/20g portion |
| Flavoured drinks | Only energy reduced or with no added sugar | 600 | 336 | 111 mg/330 ml |
| Beer and malt beverages | Only alcohol-free beer or with an alcohol content not exceeding 1.2% volume | 600 | 336 | 202 mg/600 ml |
| Beer and malt beverages | Only energy reduced beer | 25 | 14 | 8 mg/600 ml |
| Cider and perry |  | 600 | 336 | 202 mg/600 ml |
| Other alcoholic drinks including mixtures of alcoholic drinks with non-alcoholic drinks and spirits with less than 15% of alcohol |  | 600 | 336 | 101 mg/300 ml |
| Potato, cereal, flour, or starch-based snacks |  | 500 | 280 | 7 mg /25g packet |
| Table top sweetener in powder form |  | quantum satis |  |  |
| Table top sweetener in tablets |  | quantum satis |  |  |

Quantum satis means no maximum numerical level is specified and substances shall be used in accordance with good manufacturing practice, at a level not higher than is necessary to achieve the intended purpose.

**Reference:**

1. EFSA. Scientific Opinion on the re-evaluation of aspartame (E 951) as a food additive EFSA Panel on Food Additives and Nutrient Sources added to Food (ANS). EFSA Journal. 2013;11(12):3496:p1-263.
