# Supplementary material for: PKU dietary handbook to accompany PKU guidelines
Source: Orphanet J Rare Dis. 2020 Jun 30;15:171. doi: 10.1186/s13023-020-01391-y (PMC7329487; doi:10.1186/s13023-020-01391-y)
Supplement: Supplementary file 3 — Additional file 3. [file 13023_2020_1391_MOESM3_ESM.docx]

**Appendix 3: Useful strategies for persistent nausea, vomiting, dyspepsia and indigestion in PKU pregnancy**

| - Snacking on plain, carbohydrate-rich foods such as low protein toast before getting out of bed in the morning might help to reduce nausea. - Eating small food portions that are high in carbohydrate and low in fat to avoid an empty stomach, feelings of hunger and abdominal distention. - Eating a small amount of low protein bread, pasta or crackers prior to the protein substitute may help improve tolerance of the protein substitute. - Giving protein substitute chilled and in small doses up to 5 or 6 times during the day may help. It may also be better tolerated if given with extra fluid. - If the smell of liquid or powdered protein substitute is not tolerated, protein substitute tablets to replace at least one dose of protein substitute could be tried. - Drinking plenty of fluids, especially water (sipping it little and often may help prevent vomiting). - Avoid fried or spicy foods and unpleasant smells. Cold meals may be better if nausea is associated with food smells. Low protein sandwiches, vegetable wraps, rice cakes, pitta breads, and cereal bars may be helpful. - Sitting up straight during eating. Avoid lying down after a meal. - Daily exercise and good sleep are important. - To help with constipation, encourage a high fibre cereal (as part of the phenylalanine allowance). |
| --- |
