# Supplementary material for: PKU dietary handbook to accompany PKU guidelines
Source: Orphanet J Rare Dis. 2020 Jun 30;15:171. doi: 10.1186/s13023-020-01391-y (PMC7329487; doi:10.1186/s13023-020-01391-y)
Supplement: Supplementary file 4 — Additional file 4. [file 13023_2020_1391_MOESM4_ESM.docx]

**Appendix 4: The Institute of medicine (US) and National Research Council (US) Committee guidelines for pregnancy weight gain^1^**

| **Pre-pregnancy BMI ^+^** | **Total weight gain (kg)** | **Rates of Weight Gain* 2^nd^ and 3^rd^ Trimester (kg)** |
| --- | --- | --- |
|  |  | **Mean (range) in kg/week** |
| Underweight (< 18.5 kg/m^2^) | 12.5–18 | 0.51 (0.44–0.58) |
| Normal weight (18.5–24.9 kg/m^2^) | 11.5-16 | 0.42 (0.35–0.50) |
| Overweight (25.0–29.9 kg/m^2^) | 7-11.5 | 0.28 (0.23–0.33) |
| Obese (≥ 30.0 kg/m^2^) | 5-9 | 0.22 (0.17–0.27) |

**Abbreviations: BMI- Body Mass Index.**

*****Calculations assume a 0.5–2 kg weight gain in the first trimester. ^+^Adolescents should aim for weight gains at upper end of recommendations.

**Reference:**

1. Institute of Medicine (US) and National Research Council (US) Committee to Reexamine IOM Pregnancy Weight Guidelines. Weight Gain During Pregnancy: Reexamining the Guidelines. In: Rasmussen KMY, A. L., editor. Washington (DC): National Academies Press (US); 2009.
